# An Overview of Recent Developments in the Application of Antigen Displaying Vaccine Platforms: Hints for Future SARS-CoV-2 VLP Vaccines

**DOI:** 10.3390/vaccines11091506

**Published:** 2023-09-20

**Authors:** Doddy Irawan Setyo Utomo, Hamizah Suhaimi, Nor Azila Muhammad Azami, Fazren Azmi, Mohd Cairul Iqbal Mohd Amin, Jian Xu

**Affiliations:** 1 Research Center for Vaccine and Drug, Research Organization for Health, National Research and Innovation Agency (BRIN), Gedung 611, LAPTIAB, KST Habibie, Serpong, Tangerang Selatan 15314, Indonesia; dodd002@brin.go.id; 2 Centre of Drug Delivery Technology, Faculty of Pharmacy, Universiti Kebangsaan Malaysia, Jalan Raja Muda Abdul Aziz, Kuala Lumpur 50300, Malaysia; hamizahsuhaimi@gmail.com (H.S.); fazren.azmi@ukm.edu.my (F.A.); mciamin@ukm.edu.my (M.C.I.M.A.); 3 UKM Medical Molecular Biology Institute, Universiti Kebangsaan Malaysia, Kuala Lumpur 56000, Malaysia; azila_azami@ukm.edu.my; 4 Laboratory of Biology and Information Science, School of Life Sciences, East China Normal University, Shanghai 200062, China

**Keywords:** subunit vaccine, virus-like particles, VLP display, SARS-CoV-2

## Abstract

Recently, a great effort has been devoted to studying attenuated and subunit vaccine development against SARS-CoV-2 since its outbreak in December 2019. It is known that diverse virus-like particles (VLPs) are extensively employed as carriers to display various antigenic and immunostimulatory cargo modules for vaccine development. Single or multiple antigens or antigenic domains such as the spike or nucleocapsid protein or their variants from SARS-CoV-2 could also be incorporated into VLPs via either a genetic or chemical display approach. Such antigen display platforms would help screen safer and more effective vaccine candidates capable of generating a strong immune response with or without adjuvant. This review aims to provide valuable insights for the future development of SARS-CoV-2 VLP vaccines by summarizing the latest updates and perspectives on the vaccine development of VLP platforms for genetic and chemical displaying antigens from SARS-CoV-2.

## 1. Introduction

**Figure 1:**
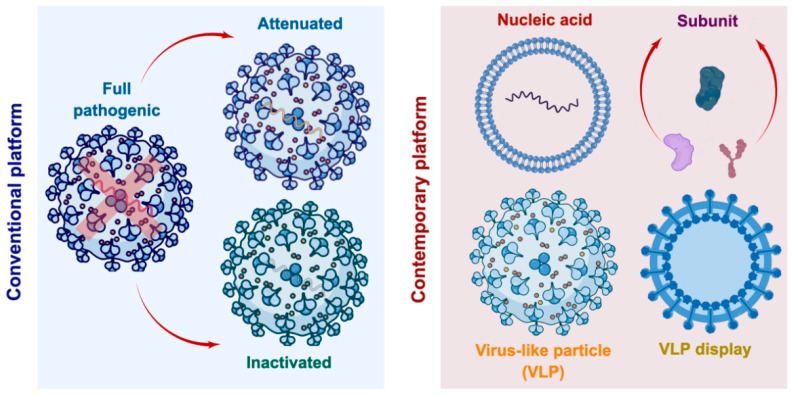
Vaccine platform strategies include conventional and contemporary platforms (Figure illustrated in Figdraw: https://www.figdraw.com, accessed on 7 October 2022).

On 11 March 2020, the World Health Organization (WHO) officially declared the coronavirus disease 2019 (COVID-19) as a global pandemic due to its rapid and widespread transmission [1]. COVID-19 is caused by the severe acute respiratory syndrome coronavirus (SARS-CoV-2), which is classified as a β-coronavirus characterized by an enveloped non-segmented positive-sense RNA [2]. In response to the SARS-CoV-2 outbreak, the WHO established the Research and Development (R&D) Blueprint to facilitate the expedited development of diagnostics, vaccines, and treatments [3]. This initiative aims to address gaps in scientific knowledge pertaining to COVID-19 and to enhance collaboration and coordination among scientists and healthcare professionals, especially in accelerating the production of an effective and safe SARS-CoV-2 vaccine [3]. The recent COVID-19 vaccine development has shifted from the conventional platform (attenuated or inactivated virus) to a contemporary platform (subunit, nucleic acid, or virus-like particles (VLPs)) (Figure 1). Over the past three decades, there has been a substantial advancement in the technology related to the preparation, bioengineering, and application of vaccines, particularly in the field of vaccinology. VLPs are protein structures that self-assemble and possess multiple subunit capabilities, closely resembling or identical to their native viruses [4]. Notably, VLPs have the potential to serve as carriers for specific antigens, epitopes, or drugs that can be displayed on their surfaces, or for encapsulating nucleic acids within VLPs to facilitate in vivo expression of desired proteins. Various studies have demonstrated that the presence of surface-displayed, densely repeated amino acids and structured VLPs can lead to a higher production of antibodies and activation of β cells [4,5,6]. Prior investigations have indicated that both enveloped and non-enveloped VLPs are commonly employed to incorporate foreign antigens, either through genetic or chemical fusion, on their surface. Both types of VLPs have been found to effectively elicit a strong immune response, with or without the use of adjuvants [7,8]. Several authors have reported that a VLP-based vaccine has demonstrated efficacy in providing protection and controlling infection against various pathogens, such as the nervous necrosis virus (NNV) and Powassan virus (POWV) [9,10]. This suggests that the use of VLP vaccines could be a promising approach in combating infectious viruses that possess antigens from different viruses or multiple strains of the same virus, such as seasonal influenza viruses and SARS-CoV-2 [5]. There have already been some FDA-approved VLP vaccines against human pathogens, such as HPV (Gardasil^®^ and Cervarix^®^), HBV (Recombivax HB^®^ and Engerix-B^®^), HEV (Hecolin^®^), and malaria (Mosquirix^TM^), and some others are currently under different stages of clinical trials [11]. However, the difficulties of VLP development may also arise during their carryover from the laboratory to the commercial scale and end-user because of the high cost, the intrinsic complexity of VLP, and potential side effects during or after vaccination [5]. In terms of VLP vaccines for SARS-CoV-2, the limited literature has shown that the immunogenic and pathologic domains of SARS-CoV-2 can be used as antigen candidates using contemporary vaccine platform strategies [12,13,14]. On the undergo of the Phase 2/3 clinical study, Yantai Patronus Biotech Co., Ltd., a company implementing the contemporary vaccine platform strategies of VLPs displaying multiple subunit antigens, is ongoing to develop the Bivalent RBD SARS-CoV-2 vaccine candidate with the name LYB001 [15]. In order to promote the advancement of current vaccine development strategies, extensive research has been conducted on the proliferation of studies pertaining to the strategy and screening of SARS-CoV-2 subunit antigens or immunological agents that are exhibited on VLPs or nanoparticles [16,17,18,19]. The objective of this review is to offer a recent update and provide insights into the contemporary SARS-CoV-2 vaccine platform, specifically in terms of the genetic and chemical display of antigens, with the aim of generating a safe, non-pathogenic, and robust immune response.

## 2. Various Protein Expression Platforms for VLPs

Over the course of several decades, extensive research has been dedicated to the examination of natural, chimeric VLPs and genetic fusion VLPs (wherein a scaffold protein is fused with an antigen) for their potential application in drug delivery for human or animal diseases [4,5,6,20,21,22]. Notably, certain investigations have successfully generated VLPs that exhibit specific antigens through both in vitro and in vivo single or co-expression methods [23,24]. Various highly effective expression host systems, such as mammalian Chinese hamster ovary (CHO) cells, yeast, bacteria (e.g., *Escherichia coli*), and insect cells, have been commercially employed for the production of recombinant VLPs [25]. The emergence of large virus-like particles (VLPs) of high quality has led to numerous studies on various expression systems. Among these systems, the baculovirus–insect cell system has garnered significant attention for the production of recombinant vaccines, and it also offers several advantages over mammalian cells, including high-level expression facilitated by natural origin promoters and post-translation modifications similar to those observed in mammalian cell expression platforms [26]. The baculovirus expression vector system (BEVS) could develop a relatively quicker method of expression via the cultured lepidopteran cell lines (e.g., Sf9 and Hi-5) or individual insect larvae such as domestic silkworm (*Bombyx mori*) and alfalfa looper (*Autographa californica*), in which recombinant bacmid DNA (*B. mori* nucleopolyhedrovirus (BmNPV) or *A. californica* multiple nucleopolyhedrovirus (AcMNPV)) is transformed or even directly injected without preparation and is being used for VLP-based vaccine production against infectious pathogens [7,20,21,22,27]. Additionally, the co-infection approach using a mixture of different recombinant baculoviruses proves advantageous for generating VLPs requiring multiple structural proteins, such as a non-enveloped virus [28,29,30,31,32,33,34,35,36]. To date, several commercial vaccines have been made in BEVS, either as subunit (e.g., Porcilis^®^ Pesti for swine fever, Flublok^®^ for the influenza virus, and Novavax vaccine for SARS-CoV-2 (NVX-CoV2373)), or VLP (e.g., Cervarix^®^ for the human papillomavirus and Porcilis^®^ PCV for Porcine circovirus type 2), while the application of *Bm*NPV in a silkworm expression system still has no licensed vaccines for human pathogens, including SARS-CoV-2 [26,37,38,39]. In contrast to other well-known protein production platforms, such as *E. coli*, yeast, and CHO cells, the establishment of a comprehensive manufacturing standard and adherence to industrial regulations, particularly GMP (good manufacturing practice), are still in progress to meet commercial requirements [40].

## 3. Genetic Display of Subunit Antigens on VLP Carriers

In order to achieve effective protein display on VLPs, a commonly utilized approach involves the expression of a viral structural protein fused with antigens, either wholly or partially (epitopes), at the N-, C-, or protrusion region, which typically depends on the structure of the VLP. This genetical approach is relatively straightforward and easy to handle, although the potential risk is that the insertion may interrupt the formation of VLPs due to the structural property of the resulting protein products. Another shortage should also be considered if the length of the VLP-forming structural protein is too short (e.g., CP3 form *Acinetobacter* phage 205 (AP205-CP3)), with which only terminal fusions of long antigens are allowed. To the best of our knowledge, several commercial VLP vaccine platforms are available, among which the hepatitis B small surface antigen (HbsAg)-derived particles and AP205-CP3-forming VLPs are studied in detail [41,42]. The rational design and engineering of original viral structural proteins for intact VLP production and display is a highly challenging yet intriguing area of research. This approach aims to achieve a higher level of mimicry of native viruses, which in turn may lead to more efficient and broader immunological protections. An example of this is the work conducted by Garg et al. [26], who successfully produced a VLP-based multivalent vaccine candidate by combining targeted antigen proteins from various viruses (Chikungunya, Japanese Encephalitis, Yellow Fever, and Zika) using a stable transformed 293 T cell line. Dealing with viruses that have multiple variants or serotypes presents several challenges for vaccine development, such as the dengue viruses, for which a commercialized vaccine is still unavailable. Several studies have explored a potential VLP-based vaccine for developing the dengue vaccine, which may comprise several serotypes [43,44]. A recent study from Utomo et al. [43] developed a dengue virus-like particle comprising a capsid-pre-membrane-envelope polypeptide from serotype 1–4 fused chemically, with which promising results in eliciting a robust immune response were seen in mice after vaccination. A study conducted by Zhai et al. [45] successfully developed a VLP-based vaccine using conserved sequence antigens (L2 epitopes) derived from different HPV types. These antigens were presented on VLPs derived from the MS2 bacteriophage, resulting in the generation of high levels of antibodies in mice. Furthermore, the vaccine demonstrated effective protection against various HPV types (HPV 16, 18, 31, 33, 45, and 58) in challenge tests, comparable to the latest version of the commercially available VLP-based multivalent HPV vaccine, Gardasil9 [45].

Meanwhile, the VLP-based vaccine is making progress in the field of anti-cancer treatment, specifically for cervical or melanoma cancer [16,46,47,48]. A study from Lizzote et al. shows that inhaling self-assembling virus-like nanoparticles from the cowpea mosaic virus (CPMV) reduces established B16F10 lung melanoma while simultaneously generating potent systemic anti-tumor immunity in the skin against poorly immunogenic B16F10 [48]. As the field of genetically displayed antigens VLP vaccine continues to develop, there remain noteworthy and pertinent issues that require attention. The foremost challenges pertain to the stability of the inter-subunit interaction genetic fusion, as the introduction of a new antigen has the potential to destabilize the VLP by interfering with the proper folding of the capsid and the antigen, resulting in a reduced production of antibodies [49]. Additionally, the complexity of the target antigen in terms of multimeric condition, formation of disulfide bonds or complex glycosylation during expression, and instability of the structure that carries different conformations in their functional role may disrupt the folding and display and, subsequently, reduce the stability of antibody complexes [49]. Notwithstanding its limitations, the research conducted by Mohsen et al. sheds light on the advantages of VLP genetic fusion, which enables the presentation of more than 60 subunits in a uniform manner on its surface, with a spacing of 5−10 nm. This characteristic enhances the immune response, as the size of the VLP is optimal for activating B cells and facilitating crosslinking between B-cell receptors [50]. Moreover, the ability of genetically fused VLP-based vaccines to serve as nanocarriers is attributed to their capacity for one-pot and versatile expression, depending on the chosen encapsulation approach. The formulation by combining several adjuvants is of utmost importance in enhancing and directing the adaptative immune response to multiply protein antigens [50].

To date, several studies related to VLP vaccines for SARS-CoV-2 have been published in recent years and are summarized in Table 1. The incorporation of amino acid substitutions, such as T941P, A944G, and K986P/V987P, is anticipated to confer spike protein with a heightened ACE2 binding ability, while also potentially stabilizing the prefusion conformation. These modifications are expected to enhance the immunogenicity of the designed SARS-CoV-2 VLP vaccine [51,52]. The stabilization of SARS-CoV-2 primarily focuses on the spike protein, and although the spike ectodomain with two proline substitutions (S-2P) has been employed to determine high-resolution structures, it is structurally unstable and difficult to produce in mammalian cells [53]. As a substitute, HexaPro can be employed instead of S-2P due to its ability to maintain the prefusion conformation, exhibit higher expression levels, and withstand both heating and freezing. Since research on epitope mapping for SARS-CoV-2 is expanding rapidly, it should be interesting to use currently available VLP platforms such as HbsAg to show and screen effective epitopes as vaccines [54,55,56].

## 4. Plug-and-Display Technology Using Covalent Binding Partners for VLP- and Nanoparticle-Based Vaccines

**Figure 2:**
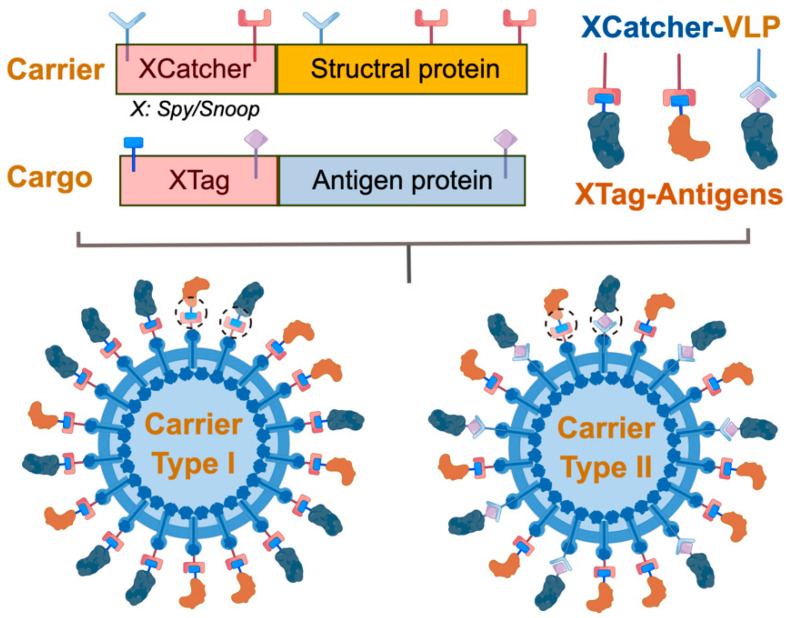
Chemical display of the SpyTag/SpyCatcher plug-and-display construction and reaction mechanism. The SpyTag-decorated VLPs with amine group are produced by fusing 13 aa SpyTag sequence with structural protein DNA sequence and are expressed in a chosen vector. Meanwhile, the SpyCatcher (138 aa) is fused with the target antigen and expressed separately in a selected vector. The SpyTag/SpyCatcher can induce spontaneous and robust protein chemistry between Lysine (Lys) and Aspartic acid (Asp) to form a covalent amide bond (dashed circles). Carrier Type I uses homogenic Xtag/XCatcher, while Carrier Type II uses heterogenic Xtag/Xcatcher. The locations of the SpyTag and SpyCatcher are flexible and should be designed and verified in a protein-dependent manner. The intermolecular isopeptide bond is formed by amide bond formation between the SpyTag/SpyCatcher-antigen and VLP, resulting in antigen display on the surface of VLP. *X* indicates Spy/Snoop. (Figure was created in Figdraw: https://www.figdraw.com, accessed on 7 October 2022).

In addition to the genetic or viral-based VLP display approaches, chemical binding, such as high affinity or covalent amino bonding between two peptides or amino acid residues, has attracted a lot of interest for developing both VLP- and nanoparticle-based vaccines [62]. One of the classical examples involves leveraging the strong affinity between surface biotinylated virus-like particles (VLPs) and antigens of interest fused with Streptavidin [63,64]. Recently, Chiba et al. engineered VLPs derived from the bacteriophage coat protein MS2 and displayed multiple copies of the spike (S) protein from SARS-CoV-2, resulting in robust protective efficacy in a hamster model [65]. The formation of a strong isopeptide bond can be achieved using the two split fibronectin domains from *Streptococcus pyogenes*, consisting of a 13 amino acid domain (SpyTag) and a 138 amino acid domain (SpyCatcher) (Figure 2) [66]. The main mechanism underlying the intermolecular isopeptide domain (amide bond) involves the release of water between the carboxyl group (Asp, Glu) and amine group (Lys), and has been demonstrated to be resilient and adaptable to diverse challenging circumstances [67]. Zakeri et al. [66] introduced a novel chemical system incorporating SpyTag/SpyCatcher, which was further enhanced by Li et al. [68] through the investigation of optimal conditions and structural characterization of its intermolecular isopeptide bond. The production of antigens and VLP nanoparticles is conducted separately and then combined to create a plug-and-display system for the antigens. This plug-and-display approach, utilizing the coat protein CP3 from bacteriophage AP205 linked with SpyTag or SpyCatcher, has been identified as a viable alternative for VLP decoration [69]. Through optimized production conditions, this method may offer time-saving advantages for future manufacturing processes. In a subsequent study, Xu et al. [70] effectively showcased the display of domain III of the E protein from four dengue virus serotypes on SpyTag/SpyCatcher in the established Spy-BEVS in vitro and in vivo. Other studies also showed that SpyTag/SpyCatcher inserted at both N- and C- terminals formed the SpyRing or cyclization conformations confer higher thermal stability and activities depending on the complexity of the protein of interest [71].

The versatility of the SpyTag/SpyCatcher plug-and-display technology is not exclusively reliant on antigen display, but has also been observed in the investigation of antigen stability. Sharma et al. [72] demonstrated the utilization of the SpyTag/SpyCatcher plug-and-display technology in assessing the structural stability of the SARS-CoV-2 protein at varying temperatures. The primary issue encountered during the formation of amide bonds is the potential asymmetry mismatch at interfaces caused by spontaneous amidation and the intricate nature of the antigen, which could impede the nano-assembly of the SpyTag/SpyCatcher system. Following this troubleshooting, Rahikainen et al. [73] conducted a study wherein they introduced a quantitative amide bond VLP coupled with a diverse symmetry of antigens, which was subsequently applied in the development of vaccines for human or animal pathogens. Additionally, an orthogonal system known as SnoopTag-SnoopCatcher, derived from the Gram-positive surface protein (*S. pneumoniae*), was introduced. This system has the ability to combine with the SpyCatcher-SpyTag and generate multiple components of protein fusions across various protein production platforms, encompassing prokaryotic and eukaryotic cells, plants, and cell-free systems [74,75,76,77]. Moreover, a combination of non-infectious adenovirus-inspired 60-mer dodecahedral VLP (ADDomer) and SpyTag/SpyCatcher must be attained by utilizing the exposed loops of the penton base protein to display a multimeric array of large antigens [78]. Adding the SpyTag peptide into the “variable loop” of the ADDomer previously used for small antigen insertion yielded a VLP with 60 potential attachment sites for complex antigens engineered with a SpyCatcher anchor [79]. Unfortunately, no licensed vaccines for SARS-CoV-2 have been developed by the SpyTag or SnoopTag chemistry, singly or orthogonally. As listed in Table 2, most research on the SpyTag/SpyCatcher plug-and-display technology in SARS-CoV-2 is still at the laboratory level, with results being encouraging [80,81,82].

The engineered protein modification for self-assembly may differ between envelope, non-envelope, and scaffold proteins. A study from Ma et al. [81] demonstrated a fusion of the SpyTag/SpyCatcher at its N-terminal nanoparticle vaccines by covalently conjugating self-assembled *Helicobacter pylori* (*H. pylori*) non-haem 24-mer ferritin to the receptor binding domain (RBD) and/or heptad repeat (HR) subunits of the SARS-CoV-2 spike (S) protein. Nanoparticle vaccines elicited stronger neutralizing antibodies and cellular immune responses than monomer vaccines. RBD and RBD-HR nanoparticle-vaccinated hACE2 transgenic mice with RBD and/or RBD-HR nanoparticles had lower viral loads in their lungs after the SARS-CoV-2 challenge. Neutralizing antibodies and cellular immune responses against other coronaviruses were also stimulated with RBD-HR nanoparticle vaccines. The lumazine synthase (LuS) scaffold protein from *Aquifex aeolicus*, which is fused with N-linked glycan and a Spy tag at the N-terminus (SpyTag-LUS/N-linked glycan), can give a solution to a problem caused by inefficiencies in nanoparticle vaccine production [82]. The SpyTag-LUS/N-linked glycan-SpyCatcher system spontaneously forms isopeptide bonds to display trimeric spike antigen SARS-CoV-2 on self-assembling nanoparticles. LuS coupled to SpyTag, and spontaneous bonds formed with SpyCatcher spike antigen SARS-CoV-2 could express effectively in a mammalian expression system when an N-linked glycan was added to the nanoparticle surface and could give decent yield and antigenicity [82]. The fusion of self-assembly protein with the SpyTag or Spy Catcher can be switched, and a few studies have shown the alternative fusion and provided a promising result [83,84,85,86,87,88]. Alternatively, a thermophilic bacteria-engineered aldolase instantaneously assembles into a hollow 36-nanometer dodecahedral cage with 60 subunits to form a nanoparticle carrier [83]. In their study, a SpyCatcher variant was developed into Spy-Catcher003 to improve reaction binding with SpyTag, provide a high immunogenic response, and demonstrate a robust neutralizing antibody response [83]. Following the same modification on the SpyCatcher variant, several studies discovered the capability of nano-scaffold of the SpyCatcher variant (SpyCatcher003-mi3) with multivalent antigen presentation and provided promising results for the vaccination strategy. This engineered aldolase VLP-type has high thermal stability and good colloidal properties due to the genetic fusing of each VLP subunit SpyCatcher003-protein through covalent isopeptide bonds [89,90,91]. Given the inherent difficulties in expressing antigen multivalent scaffolds, Guo et al. [91] conducted a study that proposed a solution to enhance expression yield by incorporating four glycosylation sites into the antigen protein. The resulting engineered protein demonstrated greater immunogenicity compared to the wild-type antigen protein. Presently, various endeavors are underway to augment the quantity and efficacy of antigen presentations on nanoparticle scaffolds, aiming to achieve a more potent defense against infections [85,86]. A protein truncation at the SpyCatcher sequence was made to increase the number of antigen presentations based on isopeptide formation and showed a promising result by exhibiting 8- to 120-fold higher neutralizing antibodies against both the pseudovirus and the authentic SARS-CoV-2 during infection [86]. In their research, Zhang et al. [85] devised a GvTagOpti/SdCatcher system based on specific proteins from Gram-positive (*Streptococcus dysgalactiae*) and Gram-negative (*Gardnerella vaginalis*) bacteria. This system demonstrated enhanced productivity and efficacy, as well as eliciting stronger immune reactions in mice when compared to the original SpyTag/SpyCatcher system. Table 2 shows pre-clinical studies and recent trends of the chemical displaying chemical displaying the SARS-CoV-2 antigen on the SpyTag/SpyCatcher plug-and-display technology able to improve the immunogenicity of mice during the vaccination by activating the T and B cells and hence eliciting the potent neutralization response. The evolution of this technology presents a promising platform for vaccine development, applicable to both human and animal disease models.

## 5. Conclusions

As of 30 March 2023, the World Health Organization (WHO) reported that there are 184 vaccines currently undergoing clinical development, with approximately 199 vaccines in the preclinical phase, in order to support global vaccine development endeavors. Additionally, the list of platforms employed for vaccine development efforts includes seven VLP-based vaccines [92]. Ongoing research is focused on the advancement of genetic and chemical displaying platforms to enhance the multivalent characteristics targeting various pathogens and their variants. Currently, a commercial malaria vaccine called Mosquirix has been developed using this platform (RTS, S), demonstrating that antigen-displaying platforms are progressing toward more commercialized vaccines [93]. Genetic antigen-displaying platforms have still not been used in the current commercial vaccine for SARS-CoV-2. On the other hand, development via the chemically displaying antigen platform revealed tough competition in vaccine research and development, as several vaccine developers were already involved in the phase 1/2 of the clinical trial for the SARS-CoV-2 vaccine [93]. More solid research incorporating diverse experimental and clinical findings is imperative to facilitate the developments of effective and evolvable VLP vaccines against these emerging pathogenic viruses.

Currently, it is widely acknowledged that the production and commercialization of VLP vaccines encounter many technical and analytical obstacles, even though the routine processes and strict industry regulations are followed. The separation and purification of artificially generated extracellular vesicles and VLPs from diverse bioreactors remain particularly challenging. To enhance the purity and efficacy of the desired VLP products, additional investigations are required to explore applicable procedures for eliminating extracellular vesicles and viruses (such as baculoviruses or lentiviruses) generated from utilized bioreactors. For instance, the utilization of fluorescent labelling and fluorescence-activated cell sorting (FACS) techniques can prove advantageous in the monitoring and optimization of both upstream and downstream processes involved in the production of virus-like particles (VLPs) [16,94,95].

## Figures and Tables

**Table 1:** Recent trends and versatility of SARS-CoV-2 subunit antigen genetically displayed on various types of VLPs.

VLP Model	SARS-CoV-2 Antigen	Results	References
SARS-CoV-2	Spike protein (S), membrane protein (M), and small envelope protein (E)	SARS-CoV-VLPs demonstrate molecular and morphological properties of native VLP	[17,57,58]
Vesicular stomatitis virus (VSVG/VSV∆G)	Chimeric spike protein receptor-binding domain (RBD) (Rhabdovirus RBD-miniSpikeminispike)	Mice immunization and its neutralizing antibodies: A single dose of VSVG/VSV∆G-miniSpikeminispike-eGFP (G) stimulates high titers of SARS-CoV-2 and protects transgenic K18-hACE2 mice from SARS-CoV-2. Homologous boost immunization of VSVG/VSV∆G-miniSpikeminispike-eGFP (G) enhanced the neutralizing antibody activity.	[59]
Newcastle disease virus (NDVLP)	Spike ectodomain (S2P)	Mice immunization and its neutralizing antibodies: S2P-NDVLP showed high spike-specific IgG titers and elicited a high humoral immune response. S2P-NDVLP can elicit substantial neutralizing activities.	[60]
Acinetobacter phage AP205	Spike protein receptor-binding domain (RBD)	Mice immunization and its neutralizing antibodies: Induce high-level IgG antibodies and are able to neutralize wild type of SARS-CoV-2.	[61]
Spike (S) protein (unmodified and modified versions), membrane glycoprotein (M), envelope (E), and nucleocapsid (N)	Spike protein (S), membrane protein (M), and small envelope protein (E)	2P with two proline substitutions (K986P and V987P), or 6P with six proline substitutions (F817P, A 892P, A899P, A942P, K986P, and V987P) to stabilize the prefusion conformation. Mice, rats, and ferrets immunization and its neutralizing antibodies: VLPs expressing the hexaproline stabilized prefusion spike antigen with Alum plus K3 CpG ODN adjuvant elicited high levels of anti-S, anti-RBD, anti-N IgG, and live virus-neutralizing antibodies.	[54]

Abbreviations: VLP: virus-like particle; VSVG/VSV∆G: vesicular stomatitis virus with glycoprotein/without; K18-hACE2: human-converting enzyme 2 expressions driven by epithelial cell cytokeratin-18; IgG: Immunoglobulin G. To avoid further conflicts, we decide not to include the results from the preprints and unpublished results.

**Table 2:** Pre-clinical studies and recent trends of the chemical displaying SARS-CoV-2 on SpyTag/SpyCatcher plug-and-display technology.

VLP Model	SARS-CoV-2 Antigen	Results	References
SpyTag-RBD/SpyTag-HR; SpyCatcher-Ferritin “RBD/HR-SpyVLP”	Spike glycoprotein receptor-binding domain (RBD)/ Heptad repeat (HR)	Mice and rhesus macaques immunization, and its neutralizing antibodies: RBD/HR-SpyVLP vaccinated hACE2 transgenic mice reduced the viral load in the lung after challenge and promoted neutralizing antibodies and cellular immune response. RBD/HR vaccinated rhesus macaques induced T and B cell responses prior to boost immunization and neutralizing antibodies.	[81]
SpyTag-LUS/ Ferritin with addition N-linked glycan: SpyCatcher-SARS-CoV-2 Spike “SARS-CoV-2 spike-LUS nanoparticles”	Spike glycoprotein	Mice immunization and its neutralizing antibodies: SARS-CoV-2spike-LUS nanoparticles significantly improved its immunogenicity with a lower dose of immunogen and elicited a potent neutralization response compared with trimeric form.	[82]
SpyTag-RBD: SpyCatcher003-mi3 “RBD-SpyVLP”	Spike glycoprotein receptor-binding domain (RBD)	Mice and pig immunization, and its neutralizing antibodies: RBD-SpyVLP induces strong ACE2-blocking, highly immunogenic, and neutralizing antibodies response in mice and pig models.	[83]
SpyTag-RBD: SpyCatcher-Ferritin “Ferritin-NP-RBD”	Spike glycoprotein receptor-binding domain (RBD)	Mice immunization and its neutralizing antibodies: Ferritin-NP-RBD enhanced and gave persistent RBD-specific antibody response. Ferritin-NP-RBD had a neutralizing effect at serum dilution and showed inhibition to the infection in vitro SARS-CoV-2.	[84]
GvTagOpti-RBD: SdCatcher-HPF “RBD-Gv/Sd NPs”	Spike glycoprotein receptor-binding domain (RBD)	Mice immunization and its neutralizing antibodies: RBD-Gv/Sd NPs give higher titers of RBD-specific immune response and neutralizing antibodies.	[85]
SpyTag-RBD: ∆N1-SpyCatcher-mi3/Ferritin/I53-50 “RBD-NPs”	Spike glycoprotein receptor-binding domain (RBD)	Mice immunization and its neutralizing antibodies: RBD-NPs successfully block the binding of RBD to ACE2 and neutralize the antibody in vitro.	[86]
SpyTag-RBD/S2∆GHR2: SpyCatcher- Ferritin SApNP/I3-01v9 SApNP (Locking domain and T-helper epitope within the SApNP “RBD-Ferritin SApNP/S2∆GHR2-I3-01v9 SApNP”	Spike glycoprotein receptor-binding domain (RBD)/ Heptad repeat-deleted glycine-capped spike(S2∆GHR2)	Mice immunization and its neutralizing antibodies: RBD-Ferritin SApNP, S2∆GHR2-NPs induced more potent neutralizing antibody compared to the control. The S2∆GHR2-NPs critically elicited required T cell immunity.	[87]
SpyTag-RBD: SpyCatcher-mi3 “RBD-SpyCatcher-mi3”	Spike glycoprotein receptor-binding domain (RBD)	Mice immunization and its neutralizing antibodies: RBD-SpyCatcher-mi3 elicited a high neutralizing antibody titer against four variants, including the delta variant and isolated SARS-CoV-2.	[88]
SpyTag003-RBD/mosaic-4a/mosaic-4b/mosaic-8: SpyCatcher003-mi3 “RBD/mosaicRBD-NPs”	Spike glycoprotein receptor-binding domain (RBD), RBD from animal betacoronaviruses	Mice immunization and its neutralizing antibodies: RBD-NPs induced cross-reactive binding and neutralizing responses, whereas mosaicRBD-NPs induced antibodies with superior cross-reactive recognition of heterologous RBDs, of either RBD-NPs or COVID-19 convalescent human plasma.	[89]
SpyTag-S/E/M/N: SpyCatcher-HBc “HBc-S/E/M/N-P-VLPs”	B cell epitopes on spike (S), envelope (E), membrane (M), nucleocapsid (N)	Mice immunization and its neutralizing antibodies: HBc-S/E/M/N-P-VLPs showed the predicted epitopes from S/E/M/N that provide wide neutralizing and different immunodominant epitopes in mice models.	[90]
SpyTag-RBD-KLH/ S-KLH: SpyCatcher-mi3 60 “RBD-mi3/S-mi3”	Spike glycoprotein receptor-binding domain (RBD) and proline-stabilized S-protein ectodomain (S)	Rodent immunization and its neutralizing antibodies: RBD-mi3 is more immunogenic and elicits potent neutralizing responses compared to S-mi3 * Glycan-modification of RBD (gRBD) has been introduced and elicits a more potent neutralizing response than RBD.	[91]
ADDomer-SpyTag: RBD-SpyCatcher “ADD-RBD”	Spike glycoprotein receptor-binding domain (RBD)	Mice immunization and its neutralizing antibodies: A multimerized RBD display led to a strong improvement in immunogenicity and elicited a higher titer of neutralizing antibodies.	[79]

* Abbreviations: NPs: nanoparticles; mi3: mutated i301 (porous dodecahedral i301 60-mer); LUS: 60-subunit *Aquifex aeolicus* lumazine synthase; HPF: *Helicobacter pylori* nonheme ferritin; GvTagOpti/Sd: derived from *Gardnerella vaginalis* and *Streptococcus dysgalactiae*; ∆N1-SpyCatcher: shortened form of SpyCatcher; SApNP/I3-01v9: self-assembling protein nanoparticles/multilayered 60-meric; HBc: hepatitis B core virus-like particle ; P: peptide; KLH: keyhole limpet hemocyanin carrier protein; ADD or ADDomer: non-infectious adenovirus-inspired 60-mer dodecahedral VLP.

## Data Availability

Not applicable.
